# Quantifying gravity-dependent and gravity-independent ventilation distribution by hyperpolarized ^129^Xe MRI in restrictive and airway diseases

**DOI:** 10.3389/fphys.2026.1835158

**Published:** 2026-08-13

**Authors:** Kunyu (Kimi) Du, David Mummy, Robert Tighe, Loretta Que, Bastiaan Driehuys, Yuh-Chin T. Huang

**Affiliations:** 1 Department of Biomedical Engineering, Duke University, Durham, NC, United States; 2 Department of Radiology, Duke University Medical Center, Durham, NC, United States; 3 Department of Medicine, Duke University Medical Center, Durham, NC, United States; 4 Department of Medical Physics, Duke University, Durham, NC, United States

**Keywords:** asthma, interstitial lung disease, MRI, pulmonary fibrosis, ventilation distribution

## Abstract

**Rationale:**

Hyperpolarized ^129^XeMRI can be used to quantify ventilation heterogeneity. The radio-frequency bias correction method has been shown to preserve the physiological ventilation gradient in healthy individuals. In this study, we tested the hypothesis that idiopathic pulmonary fibrosis (IPF) and obese asthma would have worsened gravity-dependent and gravity-independent ventilation distribution gradients.

**Methods:**

We analyzed 3-dimensional HPXeMRI ventilation images from our databank using both the conventional N4ITK and the RF bias correction methods. We divided the lung regions equally into apical/middle/basal compartments (gravity independent) and anterior/middle/posterior compartments (gravity dependent). We averaged the ventilation signals within each compartment and calculated ventilation differences (Δ $\dot{V}$) between basal and apical compartments (Δ $\dot{V}$_B-A_) and between posterior and anterior compartments (Δ $\dot{V}$_P-A_).

**Results:**

We included 10 younger (age: 23 ± 3 years), 10 older healthy subjects (age: 62 ± 7 years), 30 patients with idiopathic pulmonary fibrosis (IPF) (age: 74 ± 6 years) and 10 obese asthma patients (age: 40 ± 10 years). Younger and older healthy subjects had greater ventilation in the basal compartment (Δ $\dot{V}$_B-A_=0.20 ± 0.17 and 0.11 ± 0.15 respectively) and the posterior compartment (Δ $\dot{V}$_P-A_=0.08 ± 0.06 and 0.13 ± 0.16 respectively). Twenty-five IPF patients had positive Δ $\dot{V}$_B-A_ (0.17 ± 0.08) and Δ $\dot{V}$_P-A_ (0.11 ± 0.07). Five patients had negative Δ $\dot{V}$_P-A_ (-0.17 ± 0.09, p<0.0001) with three also having negative Δ $\dot{V}$_B-A_ (-0.04 ± 0.01, p=0.049). Six obese asthma patients had positive Δ $\dot{V}$_P-A_ (0.13 ± 0.08). Four patients had negative Δ $\dot{V}$_P-A_ (-0.11 ± 0.15, p=0.017) with two also having negative Δ $\dot{V}$_B-A_ (-0.17 and -0.09).

**Conclusions:**

With the RF depolarization bias correction method, we showed physiological ventilation gradients in healthy subjects and detected abnormal (inverse) ventilation gradients in a subgroup of patients with IPF and obese asthma, despite low ventilation defect percentage (VDP), and mild or no spirometry abnormalities. The abnormal ventilation gradients may reflect the heterogeneity of the disease that may be associated with different treatment response and prognosis. These will need to be explored in future studies with a different design including larger populations.

## Background

Pulmonary ventilation is not homogeneous, even in healthy individuals, due to gravitational and non-gravitational factors (Galvin et al., 2007; Glenny and Robertson, 2011; West, 2011). Gravity creates a hydrostatic pressure gradient in the pleural cavity such that in spontaneously breathing healthy subjects, more ventilation goes to the dependent portion of the lung. The non-gravitational factors include the asymmetric bifurcation of airways, lung weight, the shape of chest wall and the position of the diaphragm. During normal aging and in lung disease, these factors change, and thereby worsen ventilation inhomogeneity (Rodriguez-Roisin et al., 1991; Sharma and Goodwin, 2006; Teramoto and Ishii, 2007).

Some clinical and research tools have been used to assess ventilation inhomogeneity (Bayat et al., 2023; Karmali et al., 2023). Multiple inert gas elimination technique (MIGET) is the gold standard for quantifying ventilation distribution during tidal breathing. It can quantify ventilation distribution with high resolution, but the resolved ventilation distribution is not topographical. MIGET requires arterial blood gas sampling via an arterial line and mixed venous gas sampling via a pulmonary artery catheter and remains a research tool. Quantitative CT scans provide structural information in high resolution, but regional ventilation can be inferred from CT lung density (Palma et al., 2011; Vinogradskiy, 2019). The CT-based ventilation assessment carries radiation risks. Ventilation-perfusion nuclear scan can quantify regional ventilation, but the resolution is low, and it also involves radiation.

Hyperpolarized ^129^Xe MRI is a non-invasive technique that provides spatial ventilation images during breath-hold with high resolution (Stewart et al., 2022; Perron and Ouriadov, 2023). It does not involve ionizing radiation. Most imaging in the past involved 2D imaging acquisition and uses the conventional N4ITK method to correct the bias field introduced by non-uniform radiofrequency (RF) excitation across the lung volume when acquiring the image (Tustison et al., 2010). This non-uniformity introduces variations in flip angle and thus image intensity, resulting in apparent intensity gradients in the image that are not due to pulmonary physiology, but rather to factors such as RF coil architecture, body habitus, and so on. However, since the N4ITK bias field correction method is based entirely on endogenous intensity gradients in the reconstructed image, it removes real physiological ventilation gradients in addition to the desired correction for the bias field. To address this shortcoming, our group has recently introduced a method for bias field correction that estimates flip angles across the image using temporal attributes of the image acquisition (Lu et al., 2022). This “RF depolarization” bias correction method has been shown to show the expected physiological ventilation gradient in normal individuals (Lu et al., 2022).

In this study, we employ the RF bias correction method in combination with the image analysis pipeline developed at our site (He et al., 2016) to analyze 3D radial MRI images to quantify ventilation distributions in gravity-dependent and gravity-independent directions in healthy individuals and patients with idiopathic pulmonary fibrosis (IPF) and obese asthma in the supine position. This would extend the findings from Lu et al. that used the RF bias correction method in healthy individuals to patients with lung diseases. A three-compartment model was used to quantify apical-basal (gravity-independent) and anterior-posterior (gravity-dependent) ventilation gradients. The method would allow for the evaluation of the entire ventilation heterogeneity, in addition to ventilation defect percentage (VDP) that only measures the most severely affected ventilation regions. We tested the hypothesis that ventilation distribution resolved by hyperpolarized ^129^Xe MRI would be more heterogeneous in patients with IPF and obese asthma than in healthy controls.

## Methods

### Study population

We searched our database and identified older and younger individuals, patients with IPF and obese asthma who had 3D ventilation images. The healthy individuals all had normal spirometry (FEV1/FVC ≥ 0.70) and normal FEV1 (≥ 80%pred) and FVC (≥ 80%pred). The IPF patients were > 18 years old and had a clinical diagnosis of IPF confirmed by a multidisciplinary team and naïve to treatment. The asthma patients were adult subjects of age 18–65 with a physician diagnosis of late onset asthma. They had a smoking history <10 pack years with fractional exhaled nitric oxide (FeNO) < 25 ppb and a BMI ≥ 30 kg/m^2^ on regular treatment with inhaled corticosteroids (ICS) or ICS/long-acting beta-agonist (LABA) and/or long-acting muscarinic receptor antagonist (LAMA) combination medication for at least 3 months. Part of the imaging data had been analyzed with the standard bias correction method (N4ITK) and the results had been published (He et al., 2016; Rankine et al., 2020; Mummy et al., 2021). This study was a retrospective secondary analysis of images and clinical data already collected under previous primary study protocols that included normal healthy individuals, patients with IPF and obese patients with asthma. These primary studies were approved by the Duke Institutional Review Board (Pro00025110, Pro00101911, Pro00104900).

### Hyperpolarized 129Xe MRI

We retrieved 3D HPXeMRI radial ventilation images from our databank. The subjects inhaled hyperpolarized ^129^Xe from functional residual capacity (FRC) to total lung capacity (TLC) and MRI ventilation images were acquired during a 10-second breath hold. All radial Xe ventilation images were obtained with subjects in the supine position at 3T (Siemens Magnetom Trio Scanner VB19) using the following parameters: views=3600; samples/view=128; TR/TE=4.5/0.45ms; flip angle=1.5 degrees; FOV = 40cm. All 3D volumes were acquired using a randomized 3D Halton spiral radial sequence and reconstructed to a size of 128x128x128 with a constant kernel sharpness of 0.32 (Robertson et al., 2015). Images were bias-field corrected by both the N4ITK and the RF depolarization mapping (Lu et al., 2022). The N4ITK method that is a variant of the original nonparametric non-uniform normalization (N3) algorithm has become the de-facto standard owing to its ease of implementation and ability to automatically correct for bias field. N4ITK retrospectively calculates a smoothly varying bias field by iteratively sharpening the high-frequency content of the intensity distribution; it thereby attributes all low-frequency intensity variation to the bias field. However, the distribution of ventilation in the lung is heterogeneous because of gravity and the structure of the lung (non-gravity). Therefore, functional lung images have remained particularly challenging to correct, with persistent uncertainties remaining regarding how to prioritize removing bias field non-uniformities while preserving those non-uniformities that are physiologically driven. Our group has previously described the RF method using in a 2-step fashion, building on a method of Niedbalski et al (Niedbalski et al., 2019). who demonstrated multi-key reconstruction of randomized 3D radial image acquisitions (“RF depolarization mapping”). We estimated regional B1-inhomogeneity, and thereby bias field, from a single radial ventilation acquisition. We then used a larger set of such B1-inhomogeneity maps to construct a generalized flip angle map template (Studholme et al., 2004), thereby enabling bias field correction for a given coil configuration regardless of whether or not the image was acquired using a 3D radial sequence. This method retained the physiologically expected anterior–posterior ventilation gradient in healthy subjects. The subjects were scanned in a supine position.

### Imaging analysis

The 3D radial ventilation images were processed by both the traditional N4ITK method and the RF depolarization method. With the N4ITK method, we quantified the entire ventilation using linear binning based on reference distributions built from the young healthy cohort (He et al., 2016). Ventilation defects were defined as regions with ventilation signals >2 standard deviations (SDs) below the mean in the reference young health cohort. This is expressed as a percentage of total ventilation (ventilation defect percentage, VDP). Low ventilation regions were those at 1–2 SDs below the mean. High ventilation regions were > 1 SD above the mean. This method provides the information on the non-topographic ventilation distribution. The RF bias correction method was used to evaluate topographic ventilation gradients according to the algorithms previously published by our group (Lu et al., 2022).

### Compartmental analysis for topographic ventilation distribution

We divided the lung regions equally into three compartments in the apical-basal (gravity independent) and anterior-posterior (gravity-dependent) directions based on the apical-basal length of the lung (apical/middle/basal compartments) and the anterior-posterior height of the lung respectively. The model was adapted from the concept in the early work on quantifying ventilation distribution using ^133^Xe (Ball et al., 1962). This study divided the lung into upper, middle and lower long zones and showed a gravity-dependent ventilation distribution gradient in upright individuals. We averaged the ventilation signals within each compartment. We calculated differences in ventilation signals (Δ $\dot{V}$) between basal and apical compartments (Δ $\dot{V}$_B-A_) and between posterior and anterior compartments (Δ $\dot{V}$_P-A_).

### Statistical analysis

Data were expressed as mean ± SD and 95% confidence interval (CI). We compared the apical-posterior and the anterior-posterior ventilation gradients and the ventilation signals among the three compartments were analyzed by analysis of variance (ANOVA) with Tukey’s subtest for multiple comparison. P value of <0.05 was considered statistically significant.

## Results

**Figure 1:**
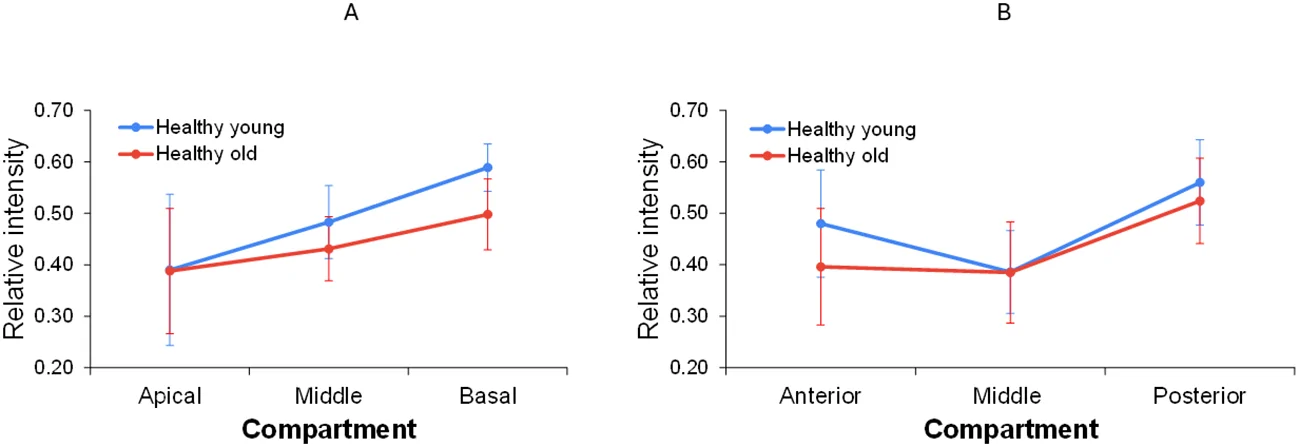
Apical-basal (gravity-independent) **(A)** and anterior-posterior (gravity-dependent) **(B)** ventilations distributions in younger healthy subjects (blue lines) and older healthy subjects (orange lines).

We included 10 younger healthy control (age: 23 ± 3 years), 10 older healthy control (age: 62 ± 7 years), 30 IPF (age: 74 ± 6 years) and 10 obese asthma patients (age: 40 ± 10 years). Their demographics and PFTs are shown in **Table 1**. VDP in younger and older healthy controls were 1.59 ± 1.54% (95% CI = 0.43,2.75) and 2.90 ± 1.87% (95% CI = 1.45,4.26) respectively (p=0.936). As a group, both younger and older healthy subjects had greater ventilation in the basal compartment (Δ $\dot{V}$_B-A_=0.199 ± 0.165, 95% CI = 0.105,0.288 and 0.110 ± 0.149, 95% CI = 0.030,0.196 respectively) and in the posterior compartment (Δ $\dot{V}$_P-A_=0.08 ± 0.06, 95% CI = 0.043,0.114 and 0.128 ± 0.159, 95% CI = 0.039,0.222 respectively). The older healthy individuals seem to have less ventilation in the basal and anterior regions, but the differences did not reach statistical significance by ANOVA, therefore, the ventilation gradients in the anterior-posterior and apical-basal directions were considered similar (**Figure 1**). One young subject had a negative Δ $\dot{V}$_B-A_ (-0.07) and Δ $\dot{V}$_P-A_ (-0.02). One older subject had a negative Δ $\dot{V}$_B-A_ (-0.21), and another subject had a negative Δ $\dot{V}$_P-A_ (-0.27).

**Table 1:** Demographics and pulmonary function tests for normal younger (N = 10), older (N = 10) subjects, patients with idiopathic pulmonary fibrosis (IPF)(N = 30) and obese asthma (N = 10).

Parameters	Healthy younger	Healthy older	IPF	Obese Asthma
Age (years)	23 (3)	62 (7)	74 (6)	40 (10)
Gender (M/F)	7/3	7/3	19/11	2/8
FVC (%pred)	102.7 (8.3)	104.1 (15.8)	78.8 (14.2)	94.0 (9.8)
FEV1 (%pred)	93.2 (9.4)	104.4 (13.6)	84.8 (16.4)	89.4 (14.7)
FEV1/FVC	0.76 (0.07)	0.78 (0.06)	0.82 (0.06)	0.76 (0.10)
DLCO (%pred)	97.3 (13.0)	95.7 (15.7)	57.2 (14.1)	-–
VDP (%)	1.59 (1.54)	2.90 (1.87)	7.33 (6.74)	2.59 (2.90)

FVC, forced vital capacity; FEV1, forced expiratory volume in 1 second; DLCO, diffusing capacity of the lungs for carbon monoxide; VDP, ventilation defect percentage. Note that DLCO was not measured in obese asthma patients.

**Figure 2:**
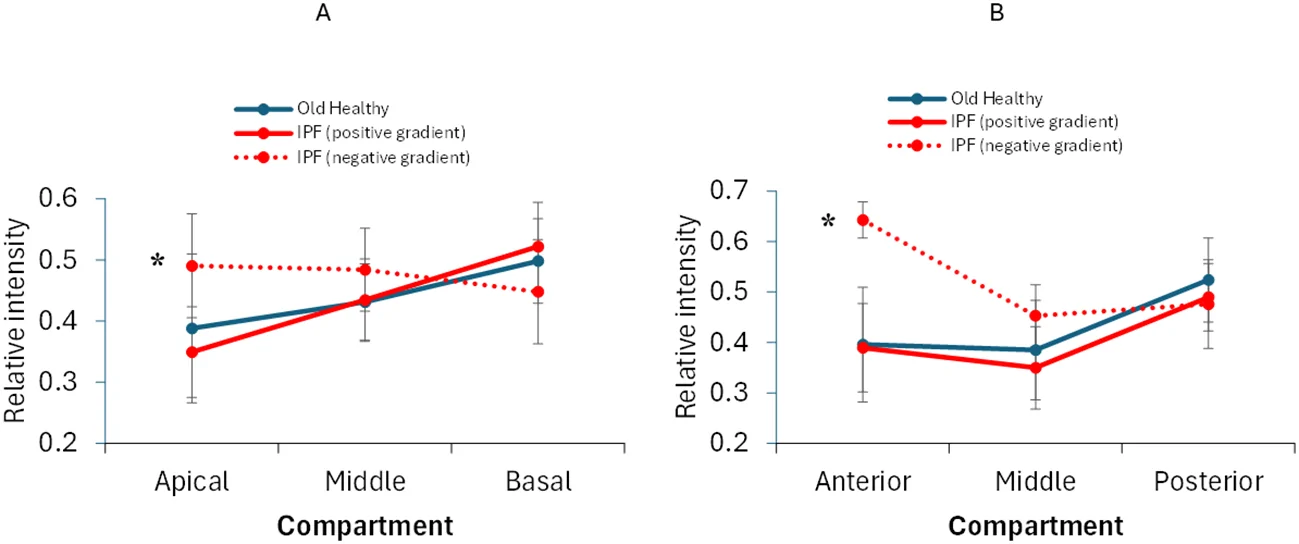
Apical-basal (gravity-independent) **(A)** and anterior-posterior (gravity-dependent) **(B)** ventilation distributions in patients with idiopathic pulmonary fibrosis with positive (orange solid lines) and negative gradients (orange dash lines) and older healthy subjects (blue lines). *p < 0.0001 vs. IPF (positive gradient).

**Figure 3:**
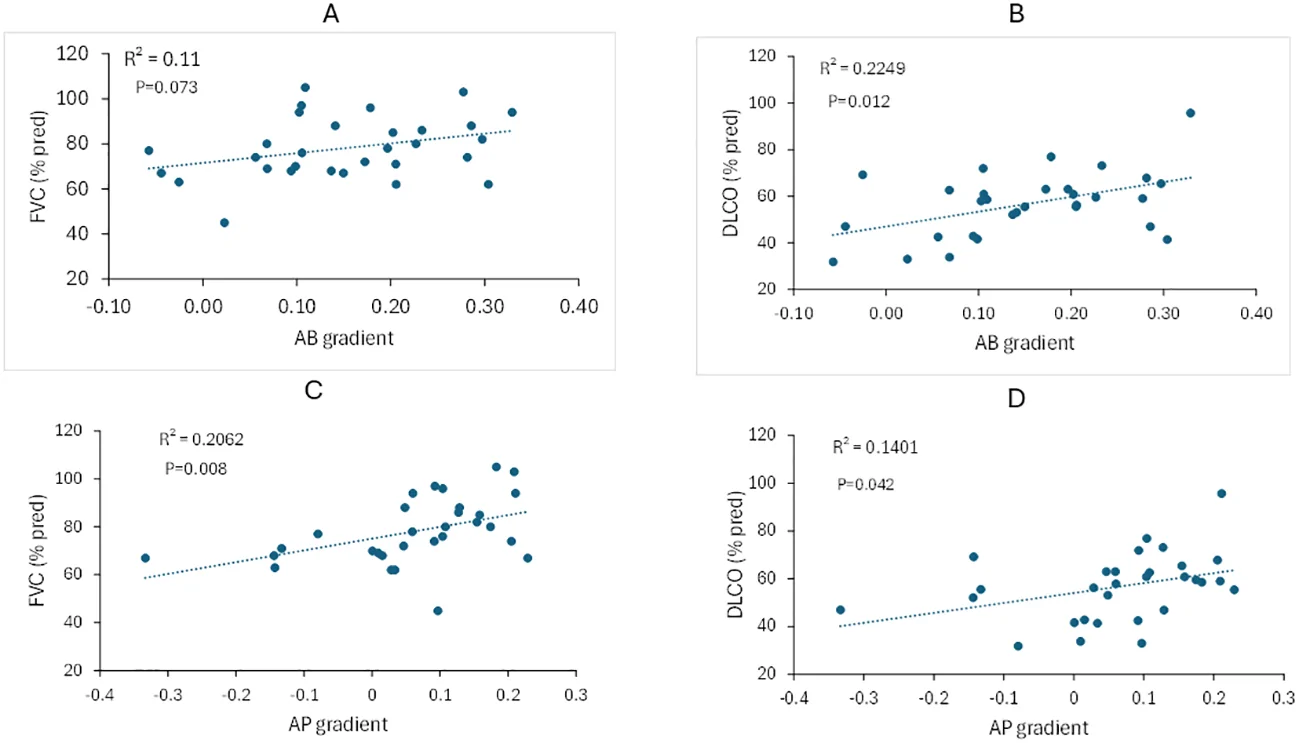
**(A)** Correlation between apical-basal (AB) gradient and FVC (%pred) (R^2^ = 0.1100, p= 0.073); **(B)** Correlation between anterior-posterior (AP) gradient and FVC (%pred) (R^2^ = 0.2062, p=0.012); **(C)** Correlation between apical-basal (AB) gradient and DLCO (%pred)(R^2^ = 0.2248, p=0.0081); **(D)** Correlation between anterior- posterior (AP) gradient and DLCO (%pred) (R^2^ = 0.1401, p=0.0416) in IPF patients.

**Figure 4:**
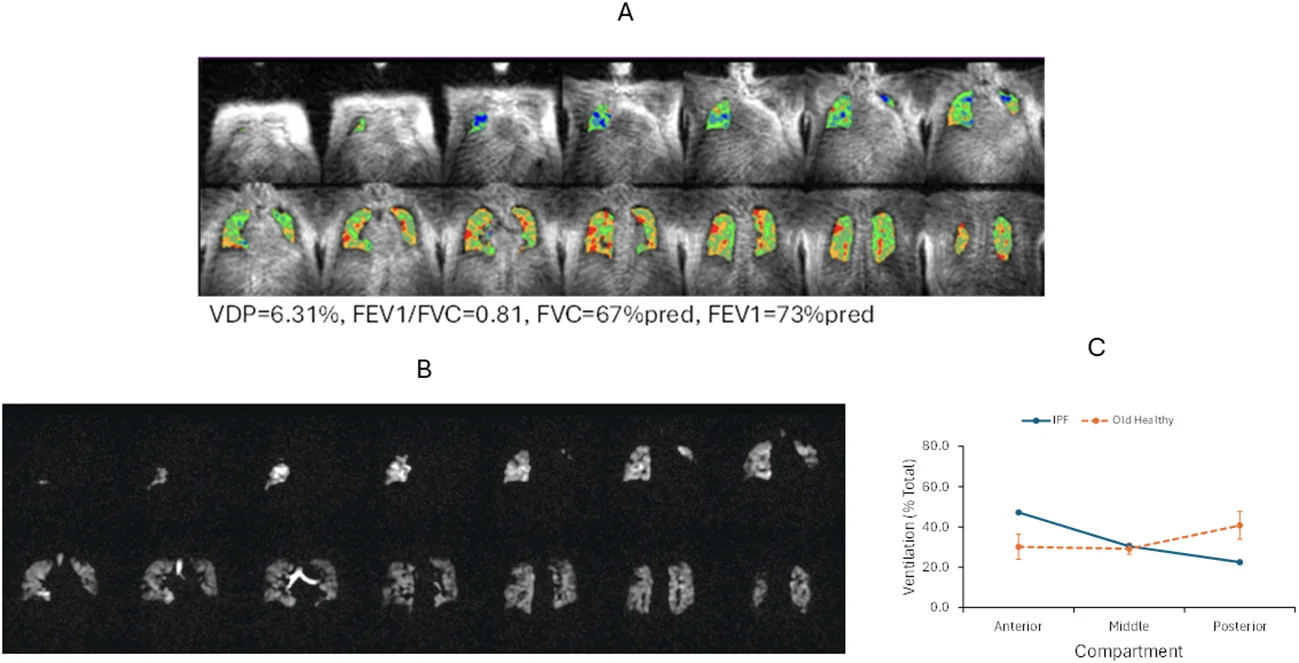
**(A)** MRI images processed by the N4ITK method of a 72 years old male patient with IPF who had both negative apical-basal and anterior-posterior ventilation distributions. Their anterior-posterior distributions are shown here. Red: regions with ventilation defect (VDP: >2 SD below the mean of a reference young health population. Orange: low ventilation regions (1–2 SD below the mean). Green: normal ventilation regions (mean ±1 SD). Blue: high ventilation regions (>1 SD above the mean). **(B)** Gray scale MRI images of the same subject processed by the RF method. **(C)** The three-compartment model of ventilation distribution in the anterior-posterior direction. Blue line: the IPF patient. Dotted orange line: older healthy control.

VDP in IPF patients at baseline were 7.33 ± 6.74% (95% CI = 4.85,10.20, p=0.0134 vs. younger healthy subjects; p=0.051 vs. older healthy subjects). Regional ventilation in IPF with positive gradient and IPF with negative gradients is shown in **Figure 2**. Among the 30 IPF patients, 25 had positive AB and AP ventilation gradients like the healthy subjects (Δ $\dot{V}$_B-A_=0.17 ± 0.08; Δ $\dot{V}$_P-A_=0.11 ± 0.07 respectively). Five patients had negative AP gradient (Δ $\dot{V}$_P-A_=-0.17 ± 0.09). Of these 5 patients, three also had negative AB ventilation gradient (Δ $\dot{V}$_B-A_=-0.04 ± 0.01). There was no difference between IPF with positive gradient and IPF with negative gradient (p=0.269) likely due to the small number of the latter. Linear regression analyses showed AB gradient (Δ $\dot{V}$_B-A_) and AP gradient (Δ $\dot{V}$_P-A_) correlated with FVC (%pred) (R^2^ = 0.1100, p= 0.073 and R^2^ = 0.2062, p=0.012 respectively) and with DLCO (%pred) (R^2^ = 0.2248, p=0.0081 and R^2^ = 0.1401, p=0.0416) (**Figure 3**). **Figure 4** shows MRI ventilation images of a 72 years old male patient with IPF who had both inverse AB and AP ventilation gradients.

**Figure 5:**
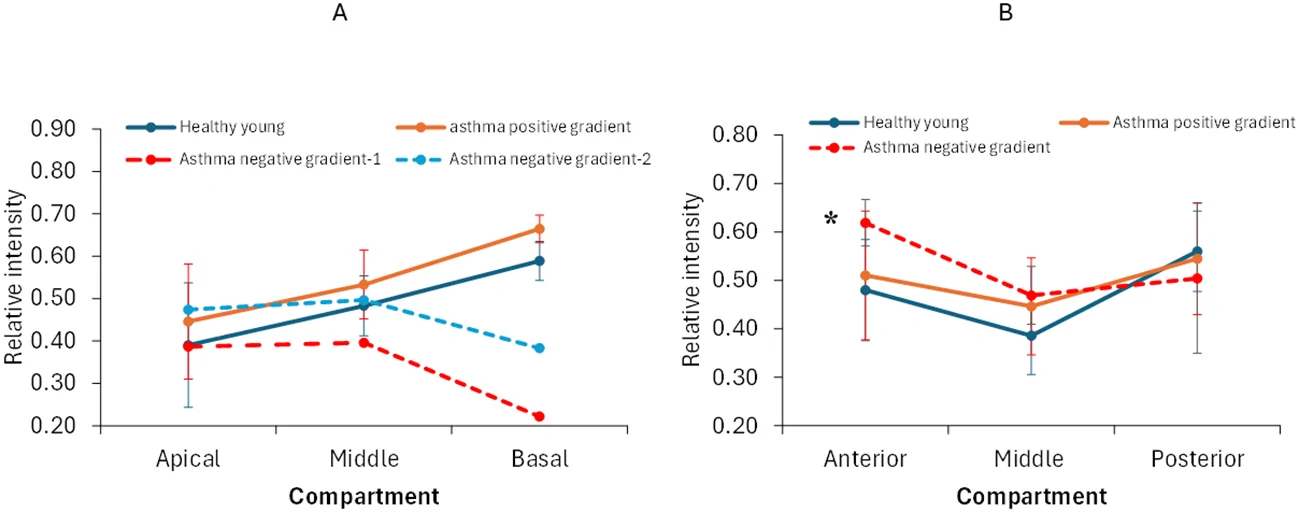
**(A)** Apical-basal (gravity-independent) and **(B)** anterior-posterior (gravity-dependent) ventilations distributions in patients with asthma with positive gradient (orange solid lines), patients with negative gradients (dash lines) and younger healthy subjects (blue lines). *p =0.017 vs. asthma patients with positive gradient.

**Figure 6:**
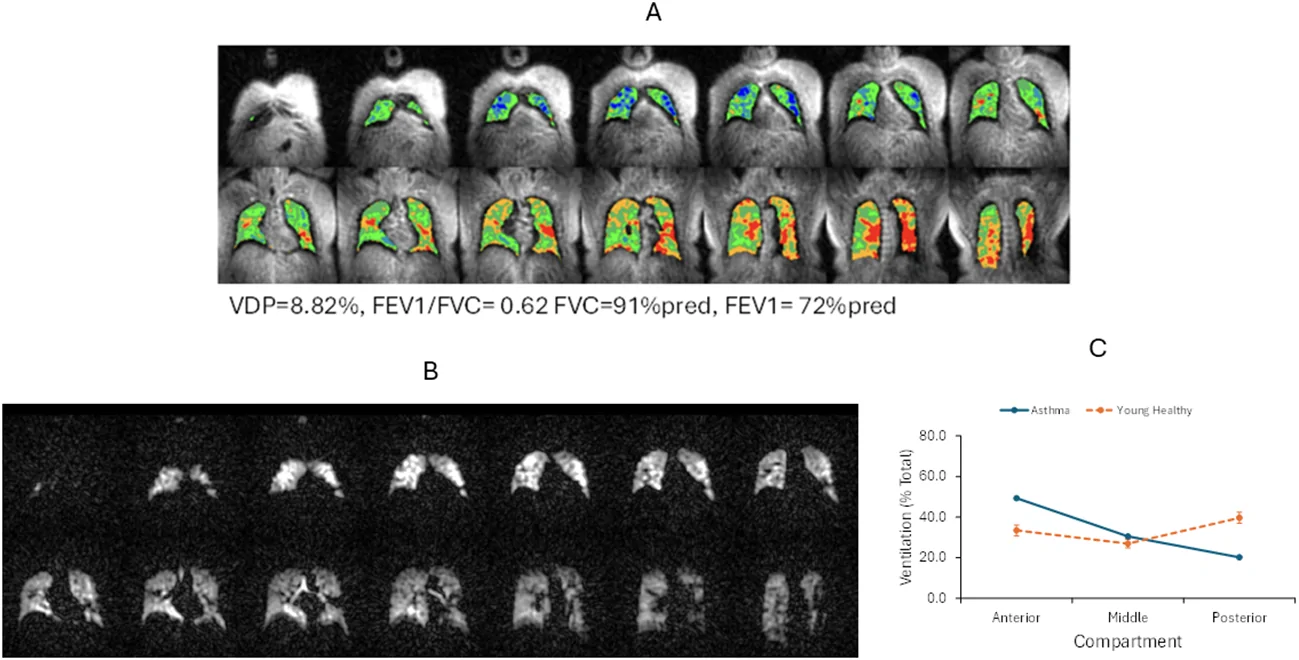
**(A)** MRI images processed by the N4ITK method of a 55 years old female patient with asthma who had both abnormal apical-basal and anterior-posterior ventilation. Distributions. Their anterior-posterior distributions are shown here. Red: regions with ventilation defect (VDP: >2 SD below the mean of a reference young health population. Orange: low ventilation regions (1–2 SD below the mean). Green: normal ventilation regions (mean ±1 SD). Blue: high ventilation regions (>1 SD above the mean). **(B)** Gray scale MRI images of the same subject processed by the RF method. **(C)** The three-compartment model of ventilation distribution in the anterior-posterior direction. Blue line: the asthma patient. Dotted orange line: younger healthy control.

In obese asthma patients, VDP was 2.59 ± 2.90% (95% CI = 0.40,4.78). As a group, the ventilation distributions were not different from those of healthy subjects (**Figure 5**). However, six patients had a positive AP ventilation gradient like the controls (Δ $\dot{V}$_P-A_=0.13 ± 0.08, 95% CI = 0.038,0.229). Their average FEV1/FVC and FEV1 were 0.80 ± 0.06%pred and 92.5 ± 12.3%pred respectively. There were 4 patients with a negative AP ventilation gradient (Δ $\dot{V}$_P-A_=-0.11 ± 0.15). Among these 4 patients, 2 also a negative AB ventilation gradient (Δ $\dot{V}$_B-A_= -0.17 and -0.09). The FEV1/FVC and FEV1 of the 4 patients were 0.71 ± 0.11 and 84.8 ± 10.5%pred, not statistically different from the 6 patients with positive ventilation gradients. **Figure 6** shows MRI ventilation images of a 55 years old female patient with asthma who had inverse AB and AP ventilation gradients.

## Discussion

In our study, we showed both younger and older healthy subjects had higher ventilation at the basal and posterior compartments. Decreased ventilation in the basal compartment and perhaps the posterior compartment has been shown by different techniques. Using electrical impedance tomography, Frerichs et al. showed less ventilation to the dependent region of the lung in the elderly (average age 73) compared to young subjects (average age 26) (Frerichs et al., 2004). Using single inhalation of ^133^Xe during breath-holding at total lung capacity, Kronenberg et al. found more ventilation in the basal lung regions in both the young and old normal subjects, but the AB ventilation gradients were similar between the young and the old groups likely due to the low resolution of ^133^Xe scan (Kronenberg et al., 1973). Cardus and Lewis showed increased low ventilation-perfusion units in the older subjects using MIGET and inert gas washout (Lewis et al., 1978; Cardus et al., 1997). Ventilation distribution resolved by MIGET, however, is non-spatial. Our results suggest that these low VA/Q units are likely in the basal and posterior regions of the lung where the perfusion is more abundant relative to ventilation. In our study, there was no statistical difference in the ventilation distribution between the younger and older healthy subjects, perhaps due to the small sample size and the simple three-compartment models we used. Since ventilation distribution is continuous, if more compartments were used to analyze the data, perhaps a difference could have been detected. This will require future studies.

A majority (25 patients or 83.3%) of our IPF patients had ventilation gradients like the older healthy subjects. This is consistent with the more prominent fibrosis in the lower lobes and the periphery of the lung resulting in decreased compliance. Patients with interstitial lung disease showed abnormal ventilation distribution resulting in increased low ventilation/perfusion regions (Wagner et al., 1976; Jernudd-Wilhelmsson et al., 1986; Agusti et al., 1991). Five IPF patients (17%) had inverse anterior-posterior ventilation gradients. Three of these patients also had an inverse apical-basal ventilation gradient. Using MIGET, Agusti et al. showed in IPF patients that ventilation distribution (LogSDv) was about 2–3 times normal, indicating increased ventilation maldistribution (Agusti et al., 1991). Their patients had low FVC. The five patients in our cohort who had inverse ventilation gradients also had reduced FVC (69.2 ± 4.7%pred) and DLCO (51.1 ± 12.1%pred). These pulmonary function abnormalities were worse than that in patients with normal gradients. Hyperpolarized ^129^XeMRI can provide spatial ventilation distribution and the ventilation gradients may be additional biomarkers for assessing the severity in patients with IPF.

Four of the 10 obese asthma patients (40%) showed inverse AB and/or AP ventilation gradients. Tzeng et al. analyzed the 2D images of ^3^He MRI in asthma patients and showed a higher ventilation heterogeneity along the craniocaudal and left-right directions (both gravity-independent) (Tzeng et al., 2009). Wagner et al. using MIGET showed 4 of 6 asymptomatic asthma had abnormal ventilation distribution with an additional mode in low VA/Q regions (Wagner et al., 1978). Wagner et al. further showed in 26 stable symptomatic asthmatic subjects that ventilation distribution was abnormal (elevated LogSDv) in 5 patients (Wagner et al., 1987). Both our study and those of Wagner et al. (1978, 1987) showed abnormal ventilation distribution in clinically stable asthma patients. Obesity can increase pleural pressure resulting from increased abdominal mass and subsequent peripheral airway occlusion and further decrease ventilation to the basal and posterior regions of the lung in a supine subject (Rabec et al., 2025). The effects of BMI on ventilation gradients will need to be investigated in future studies that include asthma patients with normal BMI. There were also more female subjects in the obese asthma group compared to other groups. Healthy female subjects were found to have more homogeneous ventilation distribution measured by electrical impedance tomography (Frerichs et al., 2024).

Our study included small sample size. The prevalence of patients with abnormal gradients will need to be validated in large future studies. In addition, the clinical implications, such as the clinical course, therapeutic responses, for the patients with abnormal gradients also need to be investigated further.

## Conclusions

In summary, using hyperpolarized ^129^Xe MRI with the RF bias correction method to analyze 3D ventilation images, we showed a subgroup of patients with IPF and obese patients with asthma had increased topographical ventilation heterogeneity, despite low VDP and mild or no spirometry abnormalities. The results are hypothesis-generating. The abnormal ventilation gradients may reflect the heterogeneity of the disease that may be associated with different treatment response and prognosis. These will need to be explored in future studies with a different design including larger populations.

## Data Availability

The raw data supporting the conclusions of this article will be made available by the authors, without undue reservation.

## References

[B1] Agusti A. G. Roca J. Gea J. Wagner P. D. Xaubet A. Rodriguez-Roisin R. (1991). Mechanisms of gas-exchange impairment in idiopathic pulmonary fibrosis. Am. Rev. Respir. Dis. 143, 219–225. doi: 10.1164/ajrccm/143.2.219 1990931

[B2] Ball W. C. Jr. Stewart P. B. Newsham L. G. Bates D. V. (1962). Regional pulmonary function studied with xenon 133. J. Clin. Invest. 41, 519–531. doi: 10.1172/jci104505 13864417 PMC290945

[B3] Bayat S. Wild J. Winkler T. (2023). Lung functional imaging. Breathe (Sheff) 19, 220272. doi: 10.1183/20734735.0272-2022 38020338 PMC10644108

[B4] Cardus J. Burgos F. Diaz O. Roca J. Barbera J. A. Marrades R. M. . (1997). Increase in pulmonary ventilation-perfusion inequality with age in healthy individuals. Am. J. Respir. Crit. Care Med. 156, 648–653. doi: 10.1164/ajrccm.156.2.9606016 9279253

[B5] Frerichs I. Braun P. Dudykevych T. Hahn G. Genee D. Hellige G. (2004). Distribution of ventilation in young and elderly adults determined by electrical impedance tomography. Respir. Physiol. Neurobiol. 143, 63–75. doi: 10.1016/j.resp.2004.07.014 15477173

[B6] Frerichs I. Handel C. Becher T. SChadler D. (2024). Sex differences in chest electrical impedance tomography findings. Physiol. Meas. 45. doi: 10.1088/1361-6579/ad5ef7 38959902

[B7] Galvin I. Drummond G. B. Nirmalan M. (2007). Distribution of blood flow and ventilation in the lung: gravity is not the only factor. Br. J. Anaesth. 98, 420–428. doi: 10.1093/bja/aem036 17347182

[B8] Glenny R. W. Robertson H. T. (2011). Spatial distribution of ventilation and perfusion: mechanisms and regulation. Compr. Physiol. 1, 375–395. doi: 10.1002/j.2040-4603.2011.tb00323.x 23737178

[B9] He M. Driehuys B. Que L. G. Huang Y. T. (2016). Using hyperpolarized (129)Xe MRI to quantify the pulmonary ventilation distribution. Acad. Radiol. 23, 1521–1531. doi: 10.1016/j.acra.2016.07.014 27617823 PMC5411263

[B10] Jernudd-Wilhelmsson Y. Hornblad Y. Hedenstierna G. (1986). Ventilation-perfusion relationships in interstitial lung disease. Eur. J. Respir. Dis. 68, 39–49. 3948933

[B11] Karmali D. Sowho M. Bose S. Pearce J. Tejwani V. Diamant Z. . (2023). Functional imaging for assessing regional lung ventilation in preclinical and clinical research. Front. Med. (Lausanne) 10, 1160292. doi: 10.3389/fmed.2023.1160292 37261124 PMC10228734

[B12] Kronenberg R. S. Drage C. W. Ponto R. A. Williams L. E. (1973). The effect of age on the distribution of ventilation and perfusion in the lung. Am. Rev. Respir. Dis. 108, 576–586. doi: 10.7326/0003-4819-76-3-413 4745254

[B13] Lewis S. M. Evans J. W. Jalowayski A. A. (1978). Continuous distributions of specific ventilation recovered from inert gas washout. J. Appl. Physiol. Respir. Environ. Exerc Physiol. 44, 416–423. doi: 10.1152/jappl.1978.44.3.416 632182

[B14] Lu J. Wang Z. Bier E. Leewiwatwong S. Mummy D. Driehuys B. (2022). Bias field correction in hyperpolarized (129) Xe ventilation MRI using templates derived by RF-depolarization mapping. Magn. Reson. Med. 88, 802–816. doi: 10.1002/mrm.29254 35506520 PMC9248357

[B15] Mummy D. G. Bier E. A. Wang Z. Korzekwinski J. Morrison L. Barkauskas C. . (2021). Hyperpolarized (129)Xe MRI and spectroscopy of gas-exchange abnormalities in nonspecific interstitial pneumonia. Radiology 301, 211–220. doi: 10.1148/radiol.2021204149 34313473 PMC8487218

[B16] Niedbalski P. J. Willmering M. M. Robertson S. H. Freeman M. S. Loew W. Giaquinto R. O. . (2019). Mapping and correcting hyperpolarized magnetization decay with radial keyhole imaging. Magn. Reson. Med. 82, 367–376. doi: 10.1002/mrm.27721 30847967 PMC6491256

[B17] Palma D. A. Van Sörnsen De Koste J. Verbakel W. F. Vincent A. Senan S. (2011). Lung density changes after stereotactic radiotherapy: a quantitative analysis in 50 patients. Int. J. Radiat. Oncol. Biol. Phys. 81, 974–978. doi: 10.1016/j.ijrobp.2010.07.025 20932655

[B18] Perron S. Ouriadov A. (2023). Hyperpolarized (129)Xe MRI at low field: Current status and future directions. J. Magn. Reson. 348, 107387. doi: 10.1016/j.jmr.2023.107387 36731353

[B19] Rabec C. Janssens J. P. Murphy P. B. (2025). Ventilation in the obese: physiological insights and management. Eur. Respir. Rev. 34(176):240190. doi: 10.1183/16000617.0190-2024 40368425 PMC12076159

[B20] Rankine L. J. Wang Z. Wang J. M. He M. Mcadams H. P. Mammarappallil J. . (2020). (129)Xenon gas exchange magnetic resonance imaging as a potential prognostic marker for progression of idiopathic pulmonary fibrosis. Ann. Am. Thorac. Soc 17, 121–125. doi: 10.1513/annalsats.201905-413rl 31593488 PMC6944348

[B21] Robertson S. H. Virgincar R. S. He M. Freeman M. S. Kaushik S. S. Driehuys B. (2015). Optimizing 3D noncartesian gridding reconstruction for hyperpolarized 129Xe MRI—focus on preclinical applications. Concepts Magn. Reson. Part A 44, 190–202. doi: 10.1002/cmr.a.21352

[B22] Rodriguez-Roisin R. Ferrer A. Navajas D. Agusti A. G. Wagner P. D. Roca J. (1991). Ventilation-perfusion mismatch after methacholine challenge in patients with mild bronchial asthma. Am. Rev. Respir. Dis. 144, 88–94. doi: 10.1164/ajrccm/144.1.88 2064144

[B23] Sharma G. Goodwin J. (2006). Effect of aging on respiratory system physiology and immunology. Clin. Interv Aging 1, 253–260. doi: 10.2147/ciia.2006.1.3.253 18046878 PMC2695176

[B24] Stewart N. J. Smith L. J. Chan H. F. Eaden J. A. Rajaram S. Swift A. J. . (2022). Lung MRI with hyperpolarised gases: current & future clinical perspectives. Br. J. Radiol. 95, 20210207. doi: 10.1259/bjr.20210207 34106792 PMC9153706

[B25] Studholme C. Cardenas V. Song E. Ezekiel F. Maudsley A. Weiner M. (2004). Accurate template-based correction of brain MRI intensity distortion with application to dementia and aging. IEEE Trans. Med. Imaging 23, 99–110. doi: 10.1109/tmi.2003.820029 14719691 PMC2291516

[B26] Teramoto S. Ishii M. (2007). Aging, the aging lung, and senile emphysema are different. Am. J. Respir. Crit. Care Med. 175, 197–198. doi: 10.1164/ajrccm.175.2.197 17200508

[B27] Tustison N. J. Avants B. B. Cook P. A. Zheng Y. Egan A. Yushkevich P. A. . (2010). N4ITK: improved N3 bias correction. IEEE Trans. Med. Imaging 29, 1310–1320. doi: 10.1109/tmi.2010.2046908 20378467 PMC3071855

[B28] Tzeng Y. S. Lutchen K. Albert M. (2009). The difference in ventilation heterogeneity between asthmatic and healthy subjects quantified using hyperpolarized 3He MRI. J. Appl. Physiol. (1985) 106, 813–822. doi: 10.1152/japplphysiol.01133.2007 19023025

[B29] Vinogradskiy Y. (2019). CT-based ventilation imaging in radiation oncology. BJR Open 1, 20180035. doi: 10.1259/bjro.20180035 33178925 PMC7592480

[B30] Wagner P. D. Dantzker D. R. Dueck R. De Polo J. L. Wasserman K. West J. B. (1976). Distribution of ventilation-perfusion ratios in patients with interstitial lung disease. Chest 69, 256–257. doi: 10.1378/chest.69.2_supplement.256-a 1248296

[B31] Wagner P. D. Dantzker D. R. Iacovoni V. E. Tomlin W. C. West J. B. (1978). Ventilation-perfusion inequality in asymptomatic asthma. Am. Rev. Respir. Dis. 118, 511–524. doi: 10.1164/ajrccm/136.3.605 707879

[B32] Wagner P. D. Hedenstierna G. Bylin G. (1987). Ventilation-perfusion inequality in chronic asthma. Am. Rev. Respir. Dis. 136, 605–612. doi: 10.1164/ajrccm/136.3.605 3631733

[B33] West J. B. (2011). A web-based course of lectures in respiratory physiology. Adv. Physiol. Educ. 35, 249–251. doi: 10.1152/advan.00042.2011 21908832

